# Breastmilk as a Multisensory Intervention for Relieving Pain during Newborn Screening Procedures: A Randomized Control Trial

**DOI:** 10.3390/ijerph182413023

**Published:** 2021-12-10

**Authors:** Hsiang-Yun Lan, Luke Yang, Chiao-Hsuan Lin, Kao-Hsian Hsieh, Yue-Cune Chang, Ti Yin

**Affiliations:** 1 School of Nursing, National Defense Medical Center, Taipei 11490, Taiwan; shinylan@mail.ndmctsgh.edu.tw; 2 Department of Social Work, Hsuan Chuang University, Hsinchu 30092, Taiwan; LHY@hcu.edu.tw; 3 Nursing Department, Tri-Service General Hospital, Taipei 11490, Taiwan; 498010365@mail.ndmctsgh.edu.tw; 4 Department of Pediatrics, Tri-Service General Hospital, Taipei 11490, Taiwan; hkh0720717@gmail.com; 5 Department of Mathematics, Tamkang University, Taipei 11490, Taiwan; ychang@math.tku.edu.tw

**Keywords:** pain, breastmilk odor, breastmilk taste, newborn infant, heel stick

## Abstract

The study aim was to explore the effects of multisensory breastmilk interventions on short-term pain of infants during newborn screening. This is a randomized controlled trial. A total of 120 newborns were recruited and assigned by randomization to one of three treatment conditions: Condition 1 = routine care (gentle touch + verbal comfort); Condition 2 = breastmilk odor + routine care; or Condition 3 = breastmilk odor + taste + routine care. Pain was scored with the Neonatal Infant Pain Scale (NIPS). Data were collected from video recordings at 1 min intervals over the 11 phases of heel sticks: phase 1, 5 min before heel stick without stimuli (baseline); phase 2 to phase 6 (during heel stick); and phase 7 to phase 11 (recovery). Generalized estimating equations compared differences in pain scores for newborns over phases among the three conditions. Compared with the routine care, provision of the odor and taste of breastmilk reduce NIPS scores during heel sticks (B = −4.36, SE = 0.45, *p* < 0.001 [phase_6_]), and during recovery (B = −3.29, SE = 0.42, *p* < 0.001 [phase_7_]). Our findings provide new data, which supports the use of multisensory interventions that include breastmilk odor and taste in combination with gentle touch and verbal comfort to relieve pain in infants undergoing newborn screening.

## 1. Introduction

The number of newborns continues to decline yearly, and the impact of the COVID-19 pandemic on the social and economic changes had contributed to an additional decline in birth rate. The World Health Organization (WHO) reported the crude newborn birth rate worldwide ((number of live births/Estimated midyear population) × 1000) was 17.96‰ in 2020, and it is estimated this rate will continue to decrease to 16.30‰ in 2030, and 14.42‰ in 2050 [1]. In Taiwan, the crude newborn birth rate was 7.01‰ in 2020, which was significantly lower than the worldwide rate; only 165,249 infants were born [2]. The vital statistics suggest that declining birthrate is a serious population problem globally, especially in Taiwan. In addition to encouraging women who are at the peak of fertility to be become pregnant and bear children, clinicians need to improve the quality of care for neonates during their early life to ensure every infant has the best opportunity to develop into a healthy adult [1].

To promote infant health, every newborn is required to receive screening for early detection of metabolic errors and diseases that can have long-term health consequences [3]. Therefore, they undergo a blood analysis 48 h after birth, which requires a heel stick to collect 5–6 microtubes (0.35–0.42 mL) of blood [4]. In addition to newborn screening, each neonate can undergo up to five addition painful procedures, including an injection of vitamin K, vaccinations, circumcision, and heel sticks to measure bilirubin or blood sugar [5]. Newborns are frequently exposed to noxious sensory stimuli, which are often ignored and undertreated by clinicians [6]. Newborn brains are continuing to develop and therefore repeated exposure to pain can alter functional connectivity, which can have a negative impact on neurological development [7], and the hypothalamic–pituitary-adrenal (HPA) axis [8]. The short-term effects of painful stimuli on newborns can alter the stability of physiological parameters, such as heart rate, oxygen saturation, and respiration [9], somatosensory thresholds [10], and neurodevelopment, which impacts brain structure, behavior, and cognitive ability [7]. The negative consequences of pain should be an incentive for clinicians to consider how to provide non-pharmacological management of pain for newborns during the routine procedures.

Analgesics may not be appropriate or effective for newborns because their pharmacokinetics and pharmacodynamics differ from those of adults and may generate adverse effects such as suppression of respiration and lethargy [11]. The negative impact of pharmacological pain management on neurodevelopment for newborns motivated us to develop interventions that can alleviate pain on a more holistic level [12]. Research suggests that pain-evoked brain activity in infants can be modulated by treatments and interventions that can generate analgesic effects [11]. Nonpharmacological supportive interventions for short-term pain in newborns include sucrose [13], nonnutritive sucking (NNS) [14], breastmilk [15], facilitated tucking [16], swaddling [17], and sensorial saturation, which uses massage, touch, smell, and voice [18]. However, the Baby-Friendly Hospital Initiative (BFHI) advocates limiting intake for newborns to breastmilk-only, unless contra-indicated by a medical condition (step 6), and, if breastfeeding, should not be offered artificial teats or pacifiers (step 9) [19]. Therefore, clinicians avoid sucrose or pacifiers for non-pharmacological interventions and instead offer either breastmilk, tucking, or swaddling.

Studies have reported that tucking [16] or swaddling [20], and breastmilk [21] can provide pain relief. The composition of essential nutrients in breastmilk benefits infants’ growth and development [22], eases digestion, enhances immunity and reduces risk of developing chronic diseases [19,23]. As a result, breastmilk combined with tucking [15,17] or swaddling [15] has been demonstrated to be effective in relieving pain in preterm infants. Breastmilk odors or breastmilk can relieve pain in preterm infants during heel sticks [24]. The mechanisms involved in pain relief from breastmilk include its sweet taste from lactose [25], odor [26], and high level of tryptophan, which increases release of endogenous opioids and beta endorphins [27]. Feeding breastmilk was as effective as 24% sucrose on relieving pain during venipuncture in preterm infants; however, sucrose was better for pain-relief in extremely preterm infants [21].

Newborns have sensory capacities allowing them to interact with the environment [28], which allows them to respond positively to olfactory stimulation associated with their mother’s breastmilk [29]. When preterm infants were simultaneously provided with the odor and taste of breastmilk, their tolerance of milk improved and they had better weight gain [30]. Term infants prefer their mother’s scents and breastmilk due to their ability to discriminate among distinct odors and tastes [30,31]. In addition, studies suggest multisensory stimulation may generate the effects of analgesia during short painful procedures [18,32]. Multisensory stimulation is composed of taste (such as oral sugar), touch (massaging the infant), and speech (attracting the infant’s attention with words). The gentle stimuli (massage and oral sugar) induce activation of descending inhibitory pathways and secretion of endorphins moderated by intermediate interneurons, which buffer the painful stimulus response in the spinal cord, called “gate control” [33]. Infants prefer their mothers’ voices to other female voices [34] and would rather listen to a female voice than a male voice. Research suggests the provision of white noise, a recording of their mother’s voice, and MiniMuffs can significantly lower pain scores, stabilize heart rates, and reduce crying time in preterm infants during heel lance [35]. Voice stimulation can also be provided during invasive procedures.

Therefore, the purpose of this study was using a combination of different sensory stimuli to examine the effects of multisensory interventions on pain responses for newborns during heel stick for newborn screening. The multisensory interventions were composed of three combinations of sensory stimuli of breastmilk odor and taste, gentle touch (GT), and verbal comfort (VC): (1) GT + VC (routine care); (2) breastmilk-odor + GT + VC; or (3) breastmilk-odor + breastmilk-taste + GT + VC. Based on the above literature and sensory mechanisms, we hypothesized the following: (1) newborns receiving breastmilk-odor + GT + VC or breastmilk-odor + breastmilk-taste + GT + VC would have lower pain scores during and after heel stick when compared with newborns receiving only GT + VC; (2) the pain-relief effects of breastmilk-odor + breastmilk-taste + GT + VC would be better than the analgesic effects of breastmilk-odor + GT + VC.

## 2. Materials and Methods

### 2.1. Design

This randomized controlled trial used a prospective repeated-measures design to compare the efficacy of three treatment conditions on pain responses in newborns before, during, and after heel stick procedures for newborn screening: Condition 1 = GT + VC (routine care); Condition 2 = breastmilk-odor + GT + VC; and Condition 3 = breastmilk-odor + breastmilk-taste + GT + VC.

### 2.2. Sample and Setting

Newborns meeting the inclusion criteria were recruited through convenience sampling from a newborn nursery of a medical center in northern Taiwan. Inclusion criteria were: (1) gestational age (GA) ≥ 37 weeks, (2) age 2–3 days, (3) birth weight ≥ 2500 g, and (4) in good health. After screening 156 newborns, 136 met the inclusion criteria. Parents of 16 newborns refused to sign the consent forms because they did not want their infants to be observed. The remaining 120 newborns were randomly assigned to one of the three multisensory conditions by a blinded statistician using a web-based blocked randomization system. The selection of newborns participating in the study is listed in Figure S1.

There were no significant differences between the participating infants and those whose parents refused participation in terms of gender, delivery type, parity, Apgar scores, and birth weight. The study power was assessed using G^*^Power version 3.1.7 with repeated-measures analysis of variance (MANOVA). The study determined effect size in accordance with the results of previous studies [17]. Based on the effect size of 0.35, a significance level of 0.05, and a study power 0.8, the total sample size must be 94. Thus, a sample size of 120 infants was sufficient.

### 2.3. Measurement of Pain in Newborns

Pain caused by heel stick newborn screening procedures was scored using the Neonatal Infant Pain Scale (NIPS), a reliable and valid assessment of pain in premature infants (GA < 37 weeks), full-term infants (GA ≥ 37 weeks), and infants at 6 weeks after delivery. The NIPS assesses pain using six indicators: facial expression, crying, breathing patterns, movements of arms, legs, and arousal [36]. Crying is assessed on a 3-point scale; the other five indicators are assessed on a 2-point scale (0–1) for pain. The total score ranges from 0 to 7, with a total score ≥ 3 indicating pain [36]. Pain was assessed by a research assistant (RA) who was well-trained in observations of video recordings of heel sticks. In this study, the inter-rater reliability of the scoring was 0.92 for baseline and ranged from 0.87 to 0.96 for phases_2–11_ across the heel stick procedures. Demographic and clinical characteristics of the newborns were obtained from review of medical and nursing charts, which included GA, gender, birth body weight, Apgar score at 1 and 5 min after birth, type of delivery, and age in days at the time the study was initiated.

After getting the approval of the institutional review board of the study setting, the first author approached parents of newborns who are compatible with the study criteria, elaborated the procedures, benefits, and harms of the study, and gained the consent from one of the parents. Then, the researchers started to collect data for each newborn.

### 2.4. Heel Sticks

An experienced senior nurse implemented heel sticks for collecting blood for newborn screening based on the standard procedures. Duration of heel stick procedures was controlled at 5 min for each newborn of the three conditions by adjusting the pressure on the heel to regulate blood flow. The heel stick procedures were divided into 11 phases: phase 1 (5 min before heel stick without stimuli [baseline, mean per minute]), phases 2–6 (the 1st, 2nd, 3rd, 4th, and 5th minutes during heel stick procedure), and phases 7–11 (recovery, from the time when the senior nurse completed blood sample collection to the end of the 5th minute after heel stick). Infants’ behavioral and physiological responses to pain were recorded with the camera lens focused on the newborn’s face, body, and legs to collect data.

### 2.5. Treatment Conditions

Thirty minutes before heel stick, newborns in all three conditions were positioned supinely and supported with rolled towels. For the group of newborns receiving routine care, the third author (a female nurse) gently touched the head (GT) and spoke softly to comfort the newborn (VC) during and after the heel sticks for newborn screening. All newborns in the intervention conditions received not only GT + VC, but also breastmilk-odor, or breastmilk-odor plus breastmilk-taste during the heel stick procedures. The multisensory interventions of breastmilk-odor plus breastmilk-taste, GT and VC were administered by the third author. In this study, most parents did not like to see their infants undergoing painful procedures. It was impossible to have the mothers hold their infants for breastfeeding during and after the heel stick procedures. Therefore, the Condition 2 and Condition 3 multisensory interventions included breastmilk from each newborn’s mother, which was expressed manually immediately when the mother woke in the morning (to reduce the influences of diet on the breastmilk flavor) and was then refrigerated and stored in the nursery. Before the newborn screening, the third author warmed up the breastmilk. Three minutes prior to the heel stick to the 5th minute of recovery, a cotton ball with the breastmilk was put near the newborn’s nostrils for breastmilk-odor. For breastmilk-taste, three milliliters of his/her own mother’s breastmilk were fed slowly through syringe dripping to the newborn’s mouth 2 min before and during the heel stick.

The interventions were consistently provided by the same intervener (the third author) who was well-trained by the principal investigator (PI) to relieve pain in newborns undergoing newborn screening procedures. The PI regularly met with the intervener and checked the procedures of the three treatment conditions to maintain consistency and integrity of the interventions.

### 2.6. Data Collection

The pain responses to the heel stick were recorded using real-time, color video recordings of the newborns across the heel stick procedures. The RA, trained by the PI, coded pain data by observing the videotapes of heel sticks based on time-triggered coding the pain indicators with 1 min intervals. Pain indicators were scored in this sequence: facial expression, crying, breathing patterns, arm and leg movements, and arousal. The inter-rater reliability was examined by coding of pain data between the PI and the RA from a random selection of 40 heel sticks to maintain the reliability of ≥0.85. The first author collected the demographic and clinical data through reviewing the participant infant’s medical and nursing charts, who knew the research purpose and plans.

### 2.7. Study Integrity

Study integrity was maintained via meetings every other week among the researchers, the RA, and senior nurse to discuss whether there had been any problems during implementation of the interventions or data collection, how issues were resolved, and to affirm that the researchers were responsible for their own task and could collect data consistently. Additionally, the PI checked whether the RA coded all the videotapes of heel sticks in random sequence and assessed the videotapes on pain scores consistently.

### 2.8. Data Analysis

We analyzed the data using the SPSS software version 24.0 (IBM, Armonk, NY, USA). Demographic and clinical data were compared using Fisher’s exact test for the categorical data and Kruskal–Wallis H-test for continuous variables. Data with continuous variables were described using means and standard deviations (SD) and categorical data were described using frequencies. We used the generalized estimating equation (GEE) method of multiple linear models to compare differences in NIPS pain scores at different phases of heel sticks among newborn infants in the three conditions, after adjusting for baseline NIPS scores. Statistical significance was defined as *p* < 0.05.

## 3. Results

### 3.1. Newborn Characteristics

The 120 full-term newborns had a mean GA of 39.13 ± 1.03 weeks. The number of females and males was similar (50.8% and 49.2%, respectively); most were born by normal spontaneous delivery (68.33%). Mean birth weight was 3100.25 ± 352.61 g. At phase_1_, newborns of the three treatment conditions were not significantly different in GA, birth weight, Apgar score, number of painful experiences before newborn screening, or interval between last feeding and heel stick. Details are shown in Table S1.

### 3.2. Pain

**Figure 1:**
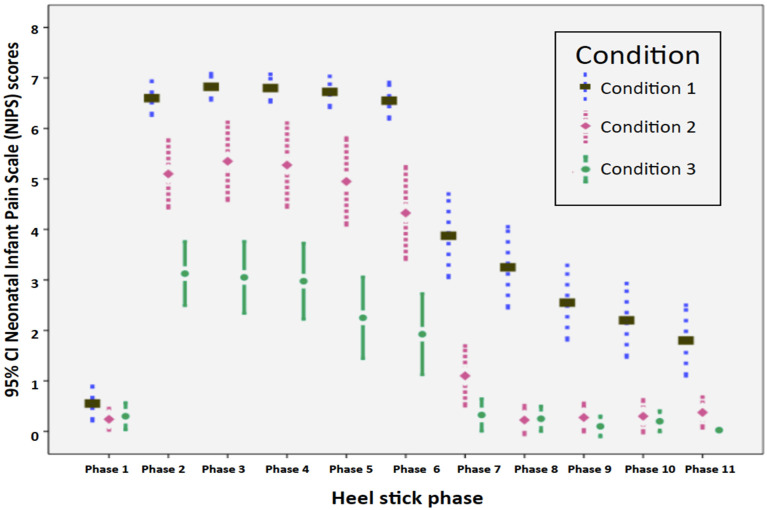
Clustered error-bar graphs. Time trends in Neonatal Infant Pain Scale (NIPS) scores of full-term infants in the three treatment conditions over 11 heel stick phases; Condition 1 (square): GT + VC; Condition 2 (diamond): Breastmilk-odor + GT + VC; Condition 3 (circle): breastmilk-odor + breastmilk-taste + GT + VC.

To simplify comparisons for the treatment conditions at each phase, the time trends of the NIPS pain scores using clustered error-bar plots are shown in Figure 1. Infants receiving Condition 2 (breastmilk-odor) and Condition 3 (breastmilk-odor + breastmilk-taste) across the heel stick procedures had significantly lower mean (standard deviation) NIPS pain scores than those receiving Condition 1 (GT + VC).

Quantitative comparisons for differences in NIPS scores among the newborns in the three treatment conditions across different phases, after adjusting for baseline scores, are shown in Table S2. Compared to Condition 1, newborns in Condition 2 showed significant lower NIPS scores during heel stick and recovery phases (phase_8_: −2.71 ± 0.41 [standard error, SE] units, *p* < 0.001; phase_11_: −1.11 ± 0.38 units, *p* < 0.01). Similarly, compared to Condition 1, newborns in Condition 3 showed significantly lower NIPS scores during heel stick and recovery phases (phase_6_: −4.36 ± 0.45 units, *p* < 0.001; phase_11_: −1.51 ± 0.38 units, *p* < 0.001). For newborns only receiving Condition 1, the changes in NIPS scores at phase_4_ were + 6.25 (±0.20) units during heel stick and + 1.25 (±0.36) units at phase_11_ during recovery (*p* < 0.01) higher than the mean NIPS scores at phase_1_.To compare the pain-relief effect between the Condition 2 and Condition 3, we used Condition 2 as the reference, and compared the effects of the different multisensory interventions (Table S3). Compared to Condition 2, newborns in Condition 3 showed significantly lower NIPS scores during heel stick and recovery phases (phase_5_: −2.75 ± 0.56 units, *p* < 0.001; phase_11_: −0.40 ± 0.17 units, *p* < 0.05). However, NIPS scores for newborns who received Condition 3 did not differ significantly from the scores for newborns receiving Condition 2 during phases 8–10.

## 4. Discussion

The study findings provide valuable knowledge about the efficacy of multisensory interventions that include breastmilk-odor or breastmilk-odor + breastmilk-taste on relieving newborn pain. The provision of three sensory stimulants (breastmilk-odor with gentle touch and verbal comfort) or the addition of a fourth stimulant (breastmilk-taste) more effectively lowered newborns’ pain scores than merely GT + VC. Condition 3, which included four sensory stimulants (breastmilk-odor + breastmilk-taste + GT + VC), had better analgesic effects than Condition 2, that included only three sensory stimulants (Breastmilk-odor + GT + VC). The study results support not only our hypotheses, but also confirm the efficacy of breastmilk-odor or breastmilk-odor + breastmilk-taste as an analgesic for newborn pain during heel sticks for newborn screening.

These findings echo those using breastmilk-taste with NNS to effectively relieve pain in preterm infants during invasive procedures [15,17]. In the study conducted by Cirik and Efe [17], 2 mL of breastmilk was provided orally in conjunction with swaddling to relieve pain during and after orogastric tube insertion procedures in preterm infants. Peng et al. [15] compared the pain-relief effects of oral breastmilk + NNS, and oral breastmilk + NNS + tucking for preterm infants during and after heel stick procedures. However, this study only provided breastmilk-odor or breastmilk-taste besides the routine care. The evidence built by this study suggests breastmilk-odor or breastmilk-odor + breastmilk-taste are effective in relieving short-term pain caused by heel stick even without the combined use of NNS or tucking.

Furthermore, some other studies report that neonates breastfed by their mothers while undergoing intrusive procedures could relieve pain caused by heel stick procedures [37,38]. Breast feeding provided by the mother is encouraged by the BFHI policies and has been validated to be effective on pain relief during short intrusive procedures [37,38]. However, our study did not use the intervention of breast feeding provided by the mother to relieve heel stick pain because most mothers were reluctant to see their infants undergoing painful procedures. Clinicians usually perform the intrusive procedures without the parents at the scene in order to reduce their stress and suffering. Therefore, this study used the multisensory intervention involved breastmilk-odor and breastmilk-taste to alleviate pain in newborns undergoing heel stick procedures to consider the mothers’ perceptions and needs.

Our results on the analgesic effects of breastmilk-odor add to the findings of several studies. This study finding is compatible with a meta-analysis reporting breastmilk-odor could lower pain scores across a procedure, compared with group not receiving sensory stimuli [24]. The findings of this study also echo a study that demonstrated the odor of breastmilk from an infant’s mother had analgesic effects during painful procedures [26]. When we compared the effects of breastmilk-odor to breastmilk-odor combined with breastmilk-taste in healthy newborns, both interventions had significant effects on pain-relief during and after the heel stick procedures. However, the provision of breastmilk-odor and breastmilk-taste was not effective on relieving preterm infants’ pain during venipuncture; the addition of NNS was needed to generate analgesic effects [39].

The study also compared the analgesic effects of breastmilk-odor (Condition 2) and breastmilk-odor+ breastmilk-taste (Condition 3) in newborns during an invasive heel stick procedure. Not only did each condition provide pain relief, but there were also significant interaction effects when Condition 2 was the reference, from phase 2 to phase 7 and during phase 11. These results suggest breastmilk-odor plus breastmilk-taste had an additive effect on relieving pain, based on the NIPS scores. Infants receiving breastmilk-odor plus breastmilk-taste recovered from pain much faster when compared with infants receiving breastmilk-odor during heel stick procedures (Figure 1). When term newborns with a mature sensory system are compared with preterm infants, the addition of olfactory and taste stimuli can generate a statistically significant increment in pain relief. Both breastmilk-odor and breastmilk-taste stimulate orogustatory receptors that activate endogenous opioid pathways to alleviate pain [25,26].

The study results add to the understanding of the ability of multisensory interventions using breastmilk delivered to stimulate smell (odor) or smell and taste (odor and taste) to provide analgesic pain relief during routine medical procedures in newborn infants. The study findings also validate the use of breastmilk without pacifiers or an artificial teat in relieving pain during intrusive procedures. In this study, the use of breastmilk-odor or breastmilk-odor plus breastmilk-taste not only alleviated pain caused by the required procedures, but also supports the policy of the BFHI to promote mothers to express breastmilk for newborns [19].

### 4.1. Clinical Implications

The study findings can act as a guide for clinicians to provide breastmilk-odor or breastmilk-odor plus breastmilk-taste to reduce newborn infant pain while receiving painful procedures. Several behaviors for new mothers could be encouraged by clinicians: skin to skin contact with their infants; allowing their newborn to suck their breast after delivery to stimulate secretion of breastmilk; and teaching parturient women how to express breastmilk for their newborns to smell and taste during the painful procedures. Some mothers might not secrete enough breastmilk in the first two days, when most painful procedures for newborns occur. Therefore, nurses should encourage mothers to express 2 mL of breastmilk dripping on a cotton ball to be placed around the newborn’s nose for breastmilk-odor and 3 mL of breastmilk for breastmilk-taste before the painful procedures. Breastmilk is natural, nutritious, and for most new mothers, easy to provide. Nurses can administer breastmilk-odor or breastmilk-taste during painful procedures without the need of a physician’s prescription. Furthermore, the use of breastmilk-odor or breastmilk-odor plus breastmilk-taste can provide an option to sucrose and NNS to alleviate newborns’ pain. This intervention would allow clinicians to support the policy of the BFHI and promote breastfeeding to benefit infants’ health and growth.

### 4.2. Study Limitations and Recommendations

The study had several limitations. First, although the RA coded all the videotapes of heel sticks in random sequence, it was not possible to maintain blinding to the conditions when evaluating the NIPS score, because the infants’ behaviors during heel sticks for the three treatment conditions were easy to discern when analyzing the videotapes. Second, whereas the sample size was sufficient, newborns were only from one medical center in Taiwan, which might limit the generalizability of the study findings. All of the newborns were full term and healthy. We do not know whether the multisensory interventions still be effective for pain relief in preterm infants with a younger GA. Future study should consider newborns with a broader range of GA, especially those infants with younger GA. The analgesic capabilities of breastmilk in infants with lower gestational ages should be further explored, as preterm infants are more likely to be deprived of maternal interactions during painful procedures. Third, the multiple sensory interventions were provided for buffering pain during heel stick, which is a short procedure. We do not know whether the multiple sensory interventions are effective for relieving pain during other procedures such as intramuscular injection or eye examination or during multiple procedures. Finally, the only outcomes we measured were the NIPS scores. Future studies might consider measuring other outcome variables such as biological outcomes (e.g., changes in heart rate, oxygen saturation, and sleep–wake states).

## 5. Conclusions

The multisensory interventions of using breastmilk-odor and breastmilk-taste effectively reduced newborns’ pain during heel stick procedures. Breastmilk-odor plus breastmilk-taste had better analgesic effects and more effectively helped newborns recover from pain than breastmilk-odor only during the heel stick procedure for newborn screening. The study results add to a growing body of evidence supporting the use of multisensory interventions with breastmilk-odor plus breastmilk-taste, GT, and VC to relieve pain in newborns undergoing routine painful procedures such as newborn screening. The evidence built by this study could guide clinicians to provide breastmilk-odor and breastmilk-taste to alleviate short pain in newborns during painful procedures.

## Data Availability

Data are available from the corresponding author on request.

## Supplementary Materials

The following are available online at https://www.mdpi.com/article/10.3390/ijerph182413023/s1, Figure S1: Flowchart of participant recruitment, Table S1: Characteristics of newborns in the three treatment conditions at birth and at time of heel stick, Table S2: Changes in pain scores for breastmilk-odor (Condition 2), and breastmilk-odor + breastmilk-taste (Condition 3) by GEE multiple linear regression with Condition 1 as reference (N = 120), Table S3: Changes in pain scores for Condition 1 (routine care) and Condition 3 (breastmilk-odor + breastmilk-taste) by GEE multiple linear regression with Condition 2 (breastmilk-odor) as reference (N = 120).
