# Supplementary material for: Breastmilk as a Multisensory Intervention for Relieving Pain during Newborn Screening Procedures: A Randomized Control Trial
Source: Int J Environ Res Public Health. 2021 Dec 10;18(24):13023. doi: 10.3390/ijerph182413023 (PMC8701293; doi:10.3390/ijerph182413023)
Supplement: Supplementary file 1 [file ijerph-18-13023-s001.zip › ijerph-1473805-supplementary.pdf]

International Journal of
Environmental Research and Public Health

MDPI

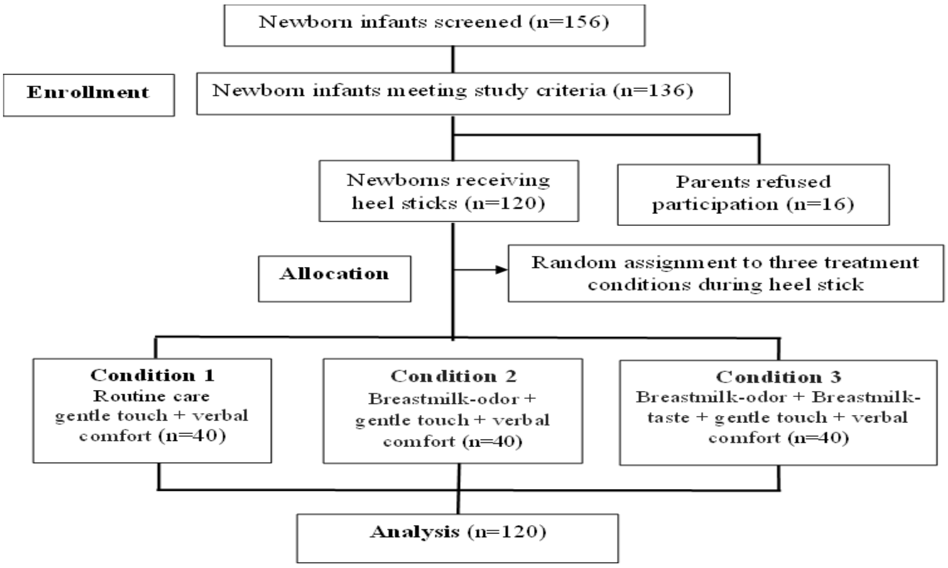

Figure S1. Flowchart of participant recruitment.

Table S1. Characteristics of newborns in the three treatment conditions at birth and at time of heel stick.

**Characteristic**	**Treatment Condition**<br>**Condition 1 (n = 40)**	**Condition 2 (n = 40)**	**Condition 3 (n = 40)**	***p***
Gestational age, weeks (M ± SD)	39.42 ± 1.00	39.07 ± 1.09	38.91±0.97	0.078[a]
Birth weight, g (M ± SD)	3121 ± 347.15	3109.38 ± 375.91	3070.38 ± 340.76	0.828[a]
Gender (n, %)				0.565[b]
Male	17 (42.5%)	22 (55.0%)	20 (50.0%)	
Female	23 (57.5%)	18 (45.0%)	20 (50.0%)	
Delivery type, n (%)				0.137[b]
Normal spontaneous delivery	32 (80)	24 (60)	26 (65)	
Caesarean delivery	8 (20)	16 (40)	14 (35)	
Parity, n (%)				0.638[b]
1	25 (62.5)	20 (50.0)	21 (52.5)	
2	10 (25.0)	16 (40.0)	13 (32.5)	
3	4 (10.0)	4 (10.0)	6 (15.0)	
4	1 (2.5)	0 (0.0)	0 (0.0)	
Age in days	3.00± 0.00	3.00± 0.00	3.00± 0.00	> 0.999 [a]
Apgar score, M ± SD				
1 min	7.88 ± 0.33	7.88 ± 0.33	7.88 ± 0.33	> 0.999 [a]
5 min	9.00 ± 0.00	8.98 ± 0.15	8.98 ± 0.15	0.604[a]
At time of heel stick (baseline)				
Time since feeding, min (M ± SD)	80.00 ± 50.33	64.75 ± 47.98	80.12 ± 44.18	0.098[a]
Number of painful experiences before newborn screening (n, %)	2.53 ± 0.55	2.40 ± 0.50	2.38 ± 0.49	0.437[b]
NIPS score (M ± SD)	0.61 ± 0.95	0.46 ± 0.79	0.42 ± 0.82	0.749[a]

Notes: M: mean; SD: standard deviation; NIPS: Neonatal Infant Pain Scale; $Condition_1$: gentle touch+ verbal comfort (routine care); $Condition_2$: breastmilk-odor + gentle touch+ verbal comfort; $Condition_3$: breastmilk-odor+ breastmilk-taste + gentle touch+ verbal comfort; $Phase_1$: baseline (no stimulation)
[a] Kruskal Wallis Test
[b]Fisher's Exact Test

Table S2. Changes in pain scores for breastmilk-odor (Condition 2), and breastmilk-odor+ breast-milk-taste (Condition 3) by GEE multiple linear regression with Condition 1 as reference (N = 120)

Variable	*B*	*SE*	Waldχ^2	*P*	95% CI Lower	95% CI Upper
**Condition effects**						
$Condition_3$ vs. $Condition_1$	-0.15	0.12	1.72	0.190	-0.38	0.08
$Condition_2$ vs. $Condition_1$	-0.23	0.12	3.77	0.052	-0.46	0.00
**Phase effects**						
$Phase_{11}$ vs. $Phase_1$	1.25	0.36	11.94	0.001	0.54	1.96
$Phase_{10}$ vs. $Phase_1$	1.65	0.36	21.14	<0.001	0.95	2.35
$Phase_9$ vs. $Phase_1$	2.00	0.35	32.07	<0.001	1.31	2.69
$Phase_8$ vs. $Phase_1$	2.70	0.39	47.56	<0.001	1.93	3.46
$Phase_7$ vs. $Phase_1$	3.32	0.41	65.07	<0.001	2.52	4.13
$Phase_6$ vs. $Phase_1$	6.00	0.24	636.29	<0.001	5.53	6.46
$Phase_5$ vs. $Phase_1$	6.17	0.21	867.63	<0.001	5.76	6.58
$Phase_4$ vs. $Phase_1$	6.25	0.20	946.22	<0.001	5.85	6.65
$Phase_3$ vs. $Phase_1$	6.27	0.21	919.26	<0.001	5.87	6.68
$Phase_2$ vs. $Phase_1$	6.05	0.22	728.17	<0.001	5.61	6.49
**Interaction effects**						
**Condition 3**						
$Condition_3 \times Phase_{11}$	-1.51	0.38	15.60	<0.001	-2.26	-0.76
$Condition_3 \times Phase_{10}$	-1.74	0.38	20.76	<0.001	-2.48	-0.99
$Condition_3 \times Phase_9$	-2.19	0.37	35.20	<0.001	-2.91	-1.46
$Condition_3 \times Phase_8$	-2.74	0.41	44.36	<0.001	-3.54	-1.93
$Condition_3 \times Phase_7$	-3.29	0.42	59.85	<0.001	-4.12	-2.55
$Condition_3 \times Phase_6$	-4.36	0.45	93.95	<0.001	-5.24	-3.48
$Condition_3 \times Phase_5$	-4.21	0.43	94.38	<0.001	-5.06	-3.36
$Condition_3 \times Phase_4$	-3.56	0.41	75.80	<0.001	-4.36	-2.76
$Condition_3 \times Phase_3$	-3.51	0.39	79.74	<0.001	-4.28	-2.74
$Condition_3 \times Phase_2$	-3.21	0.39	68.20	<0.001	-3.97	-2.45
**Condition 2**						
$Condition_2 \times Phase_{11}$	-1.11	0.38	8.59	0.003	-1.85	-0.37
$Condition_2 \times Phase_{10}$	-1.59	0.37	18.00	<0.001	-2.32	-0.85
$Condition_2 \times Phase_9$	-1.96	0.39	25.69	<0.001	-2.27	-1.20
$Condition_2 \times Phase_8$	-2.71	0.41	44.78	<0.001	-3.51	-1.92
$Condition_2 \times Phase_7$	-2.46	0.48	26.32	<0.001	-3.40	-1.52
$Condition_2 \times Phase_6$	-1.91	0.50	14.36	<0.001	-2.90	-0.92
$Condition_2 \times Phase_5$	-1.46	0.46	9.95	0.002	-2.37	-0.55
$Condition_2 \times Phase_4$	-1.21	0.45	7.30	0.007	-2.09	-0.33
$Condition_2 \times Phase_3$	-1.16	0.43	7.38	0.007	-2.00	-0.32
$Condition_2 \times Phase_2$	-1.19	0.39	9.45	0.002	-1.95	-0.43
**Baseline pain score**	0.54	0.10	30.66	<0.001	0.35	0.73

Notes: SE: standard error; CI: confidence interval; $Condition_1$: gentle touch+ verbal comfort (routine care); $Condition_2$: breastmilk-odor + gentle touch+ verbal comfort; $Condition_3$: breastmilk-odor + breastmilk-taste + gentle touch+ verbal comfort; $Phase_1$: baseline (no stimulation); $Phase_2$: 1st minute during heel stick; $Phase_3$: 2nd minute during heel stick; $Phase_4$: 3rd minute during heel stick; $Phase_5$: 4th minute during heel stick; $Phase_6$: 5th minute during heel stick; $Phase_7$: 1st minute after heel stick; $Phase_8$: 2nd minute after heel stick; $Phase_9$: 3rd minute after heel stick; $Phase_{10}$: 4th minute after heel stick; $Phase_{11}$: 5th minute after heel stick; Condition effects: comparison of the mean NIPS scores at $phase_1$ ($Condition_2$ versus $Condition_1$, $Condition_3$ versus $Condition_1$); phase effects: the changes (from $phase_1$) of the mean NIPS scores across the phases in the $Condition_1$ (phase2, 3, 4, 5, 6, 7, 8, 9, 10, 11, respectively, versus $phase_1$); Interaction effects: comparisons of differences in the changes

(from $phase_1$) of the mean NIPS scores between $Condition_3$ (or $Condition_2$) and $Condition_1$ across the phases.

Table S3. Changes in pain scores for Condition 1 (routine care), and Condition 3 (breastmilk-odor+ breastmilk-taste) by GEE multiple linear regression with Condition 2 (breastmilk-odor) as reference (N = 120)

Variable	*B*	*SE*	Waldχ^2	*P*	95% CI	
					Lower	Upper
**Condition effects**						
$Condition_3$ vs. $Condition_2$	0.08	.093	0.71	0.398	-0.10	0.26
$Condition_1$ vs. $Condition_2$	0.23	.119	3.77	0.052	-0.00	0.46
**Phase effects**						
$Phase_{11}$ vs. $Phase_1$	0.14	0.11	1.50	0.220	-0.08	0.35
$Phase_{10}$ vs. $Phase_1$	0.06	0.11	0.31	0.578	-0.15	0.27
$Phase_9$ vs. $Phase_1$	0.04	0.16	0.05	0.827	-0.28	0.35
$Phase_8$ vs. $Phase_1$	-0.02	0.11	0.02	0.888	0.22	0.19
$Phase_7$ vs. $Phase_1$	0.86	0.25	12.18	<0.001	0.38	1.34
$Phase_6$ vs. $Phase_1$	4.09	0.45	84.22	<0.001	3.21	4.96
$Phase_5$ vs. $Phase_1$	4.71	0.41	129.66	<0.001	3.90	5.52
$Phase_4$ vs. $Phase_1$	5.04	0.40	158.40	<0.001	4.25	5.82
$Phase_3$ vs. $Phase_1$	5.11	0.37	185.99	<0.001	4.38	5.84
$Phase_2$ vs. $Phase_1$	4.86	0.31	238.68	<0.001	4.24	5.48
**Interaction effects**						
**Condition 3**						
$Condition_3 \times Phase_{11}$	-0.40	0.17	5.69	0.017	-0.73	-0.07
$Condition_3 \times Phase_{10}$	-0.15	0.17	0.79	0.374	-0.48	0.18
$Condition_3 \times Phase_9$	-0.22	0.19	1.36	0.243	-0.60	0.15
$Condition_3 \times Phase_8$	-0.02	0.16	0.02	0.883	-0.35	0.30
$Condition_3 \times Phase_7$	-0.82	0.27	9.55	0.002	-1.35	-0.30
$Condition_3 \times Phase_6$	-2.45	0.59	17.42	<0.001	-3.60	-1.30
$Condition_3 \times Phase_5$	-2.75	0.56	23.96	<0.001	-3.85	-1.65
$Condition_3 \times Phase_4$	-2.35	0.54	19.26	<0.001	-3.40	-1.30
$Condition_3 \times Phase_3$	-2.35	0.50	21.86	<0.001	-3.33	-1.36
$Condition_3 \times Phase_2$	-2.02	0.45	20.48	<0.001	-2.90	-1.15
**Condition 1**						
$Condition_1 \times Phase_{11}$	1.11	0.38	8.69	0.003	0.37	1.85
$Condition_1 \times Phase_{10}$	1.59	0.37	18.00	<0.001	0.85	2.32
$Condition_1 \times Phase_9$	1.96	0.39	25.69	<0.001	1.20	2.72
$Condition_1 \times Phase_8$	2.71	0.41	44.78	<0.001	1.92	3.51
$Condition_1 \times Phase_7$	2.46	0.48	26.32	<0.001	1.52	3.40
$Condition_1 \times Phase_6$	1.91	0.50	14.36	<0.001	0.92	2.90
$Condition_1 \times Phase_5$	1.46	0.46	9.95	0.002	0.55	2.37
$Condition_1 \times Phase_4$	1.21	0.45	7.30	0.007	0.33	2.09
$Condition_1 \times Phase_3$	1.16	0.43	7.38	0.007	0.32	2.00
$Condition_1 \times Phase_2$	1.19	0.39	9.45	0.002	0.43	1.95
**Baseline pain score**	0.54	0.10	30.66	<0.001	0.35	0.73

Notes: SE: standard error; CI: confidence interval; $Condition_1$: gentle touch+ verbal comfort (routine care); $Condition_2$: breastmilk-odor + gentle touch+ verbal comfort; $Condition_3$: breastmilk-odor + breastmilk-taste + gentle touch+ verbal comfort; $Phase_1$: baseline (no stimulation); $Phase_2$: 1st minute during heel stick; $Phase_3$: 2nd minute during heel stick; $Phase_4$: 3rd minute during heel stick; $Phase_5$:

4th minute during heel stick; $Phase_6$: 5th minute during heel stick; $Phase_7$: 1st minute after heel stick; $Phases_8$: 2nd minute after heel stick; $Phase_9$: 3rd minute after heel stick; $Phase_{10}$: 4th minute after heel stick; $Phase_{11}$: 5th minute after heel stick; Condition effects: comparison of the mean NIPS scores at $phase_1$ ($Condition_3$ versus $Condition_2$, $Condition_1$ versus $Condition_2$); phase effects: the changes (from $phase_1$) of the mean NIPS scores across the phases in the $Condition_2$ (phase2, 3, 4, 5, 6, 7, 8, 9, 10, 11, respectively, versus $phase_1$); Interaction effects: comparisons of differences in the changes (from $phase_1$) of the mean NIPS scores between $Condition_3$ (or $Condition_1$) and $Condition_2$ across the phases.
